# *Pseudomonas fluorescens *biofilms subjected to phage phiIBB-PF7A

**DOI:** 10.1186/1472-6750-8-79

**Published:** 2008-10-27

**Authors:** Sanna Sillankorva, Peter Neubauer, Joana Azeredo

**Affiliations:** 1 IBB-Institute for Biotechnology and Bioengineering, Centre of Biological Engineering, Universidade do Minho, Campus de Gualtar 4710-057, Braga, Portugal; 2 Bioprocess Engineering Laboratory, Department of Process and Environmental Engineering and Biocenter Oulu, University of Oulu, P.O. Box 4300, FI-90014 Oulu, Finland

## Abstract

**Background:**

*Pseudomonas fluorescens *is an important food spoilage organism, usually found in the form of biofilms. Bacterial biofilms are inherently resistant to a variety of antimicrobial agents, therefore alternative methods to biofilm control, such as bacteriophages (phages) have been suggested. Phage behavior on biofilms is still poorly investigated and needs further understanding. Here we describe the application of phage ϕIBB-PF7, a newly isolated phage, to control *P. fluorescens *biofilms. The biofilms were formed under static or dynamic conditions and with or without renewal of medium.

**Results:**

Conditions for biofilm formation influenced the feature of the biofilm and the morphology of *P. fluorescens*. Biomass removal due to phage activity varied between 63 and 91% depending on the biofilm age and the conditions under which the biofilm had been formed and phages applied. Removal of the biofilm by phage treatment was faster in younger biofilms, but the same number of surviving cells was detected in all tested biofilms, after only 4 h of treatment, even in older biofilms. Under static conditions, a 3 log higher number of phage progeny remained either inside the biofilm matrix or attached to the substratum surface than under dynamic conditions, pointing to the importance of experimental conditions for the efficacy of phage entrapment into the biofilm.

**Conclusion:**

Phage ϕIBB-PF7A is highly efficient in removing *P. fluorescens *biofilms within a short time interval. The conditions of biofilm formation and applied during phage infection are critical for the efficacy of the sanitation process. The integration of phages into the biofilm matrix and their entrapment to the surface may be further beneficial factors when phage treatment is considered alone or in addition to chemical biocides in industrial environments where *P. fluorescens *causes serious spoilage.

## Background

*Pseudomonas fluorescens *is a spoilage causing bacterium present in a variety of food related environments. In dairy industry, *P. fluorescens *is one of the most commonly isolated psychrotrophic bacteria that dominate the microflora of raw or pasteurized milk at the time of spoilage [1-6]. This spoilage ability is mostly due to the capability of producing heat-stable extracellular lipases, proteases and lecithinases that survive the thermal processing steps [2,7-9]. This bacterium is also one of the three most predominantly isolated bacteria associated with spoilage of fresh poultry and reports of spoilage due to *P. fluorescens *date since the 1930s [10-13]. More recently, some studies have revealed that some strains of *Pseudomonas *can increase the colonization of inert surfaces by *Listeria monocytogenes *[14] and/or protect this pathogenic bacterium from disinfectants[15]. There are also studies reporting spoilage of refrigerated foods with *P. fluorescens*, in particular of refrigerated meat products [16-18]. The contamination of the meats results in changes in appearance and odour during prolonged storage. Furthermore, *P. fluorescens *is also recognized to be a model organism for biofilm studies as it can easily form biofilms in different laboratory simulators [19-21].

The interest in applied bacteriophage research has increased during recent years mainly due to positive results obtained with phage therapy applied to animals [22-25]. Also phage application to certain meat products was allowed, since August 2006, by the United States Food and Drug Administration (FDA) in order to control *Listeria monocytogenes *[26]. Moreover, there is an increase in the number of patents of application of phages to control pathogenic bacteria in industrial environments and foodstuffs [27-30]. The idea in this area is to either keep the pathogen propagation limited by the phages over long times, e.g. by insertion of phages in surface layers (e.g. [31]) or to apply phages at different stages of production and processing to reduce food product contamination at that point or to protect against contaminations at subsequent points, which can be performed also in combination of sterilizing chemical agents as long as these agents do not reduce the biological activity of the phages [30].

Biofilm phenomena have been studied over many years and it is generally recognized that this bacterial lifestyle on surfaces is dominant. Although many studies involve phage infection of cells, most of them only consider planktonic bacteria. Bacteria attached to surfaces have totally different characteristics such as they are embedded in a matrix composed of exopolysaccharides, proteins and nucleic acids and the cells represent different growth stages. As phage infection and life cycle generally strongly depend from the growth stage of the host bacterium (see e.g. [32,33]) the treatment of slowly growing cells in biofilms is a challenge. Some studies have already been made regarding the application of phages to eradicate bacteria in the form of biofilm [31,34-39] nonetheless more understanding of phage action in biofilm influenced by age and formation conditions is still required.

In a previous work with biofilms [38] it was already showed that under optimal conditions phage ϕS1 could infect well *P. fluorescens *biofilms. This was a study performed with a culture collection host and phage system that had been isolated from soil. Here a *P. fluorescens *isolated from a dairy industry was used. The bacterium was isolated from a biofilm present on the stainless steel teatcup shell which indicates that this isolate would easily form biofilms on stainless steel slides. The aim of this work was to study the application of the recently isolated and characterized T7-like bacteriophage ϕIBB-PF7A [40] to control *P. fluorescens *biofilms. Aside from a very high efficiency of this phage in biofilm removal, different conditions applied for biofilm formation and during phage application proved their influence on the time kinetics of the phage absorption and biofilm removal process. Interestingly however, the single species model biofilm was efficiently removed under all conditions which suggests phage ϕIBB-PF7A to be a superior sanitation agent.

## Results

### Biofilms formed under different conditions

In this work, *P. fluorescens *biofilms were allowed to form onto stainless steel (SS) slides, which is a common substratum used in the dairy industry. Three different experimental approaches for biofilm formation were applied: (i) static conditions, i.e. no shaking applied, with media renewal every 12 h (SR), (ii) dynamic conditions, i.e. incubation on an orbital shaker, with media renewed every 12 h (DR), and (iii) dynamic conditions without renewal of cultivation medium (DNR) (see Methods for description of the different conditions). Also, different biofilm ages were studied ranging from 24 up to 168 hours.

**Figure 1:**
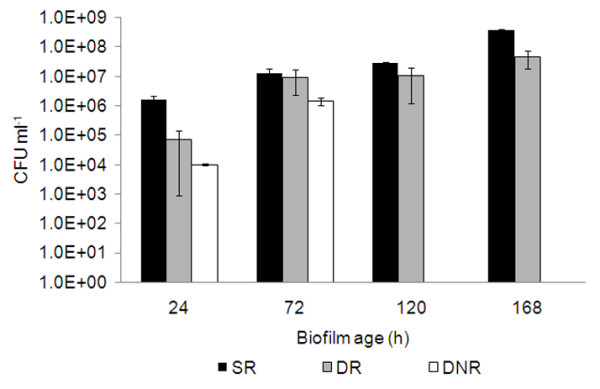
**Number of biofilm cells present on stainless steel slides.***P. fluorescens *biofilms were formed during different times on stainless steel slides under different conditions: static with media renewal (SR), dynamic with media renewal (DR), dynamic non-renewal of media (DNR). For all experiments n = 6. Error bars indicate standard deviations.

The number of cells present on SS slides was assessed after different periods of biofilm formation (Fig. 1). Lowest cell counts, detected as colony forming units after removal of the cells from the substratum, were obtained from biofilms which were incubated under shaking conditions (dynamic conditions) with an additional renewal of the cultivations medium. Dependent on the incubation period, 10 to 100 times higher cell numbers were detected from coupons which were incubated without any turbulence, i.e. under static (SR) conditions (Fig. 1). Interestingly, the difference in the number of colony forming units between the different conditions decreased with the time of incubation.

Additionally to the determination of colony forming units, also the dry weight of the biofilms was analyzed. These dry weight measurements cannot be directly correlated with viable cell numbers (Table 1). For example, the viable cell counts obtained from biofilms which were cultivated for 72 h under static or dynamic conditions (DR and SR) were practically identical, but the determined dry weight was clearly different. This difference in dry weigh is most likely due to different amounts of EPS matrix present in these different biofilms.

**Table 1:** Biomass before and after 4 h of phage application to different biofilms and biomass removal percentages

	Biofilm age (h)	Biomass beginning (0 h) (± SD)	Biomass end (4 h phage) (± SD)	Biomass removal (%)
SR	24	8.50 (0.71)	1.50 (1.08)	82.35
	72	12.50 (3.54)	3.00 (1.73)	76.00
	120	13.67 (4.73)	5.00 (1.15)	63.42
	168	14.67 (3.79)	4.00 (3.06)	72.73

DR	24	5.00 (1.41)	1.00 (0.00)	80.00
	72	7.50 (0.71)	0.67 (0.58)	91.07
	120	8.67 (0.50)	2.00 (1.46)	76.93
	168	12.50 (3.21)	4.33 (1.15)	65.36

DNR	24	7.33 (2.08)	2.00 (1.52)	72.71
	72	11.00 (1.73)	3.00 (2.06)	72.72

SD means standard deviations from 6 different SS slides.

**Figure 2:**
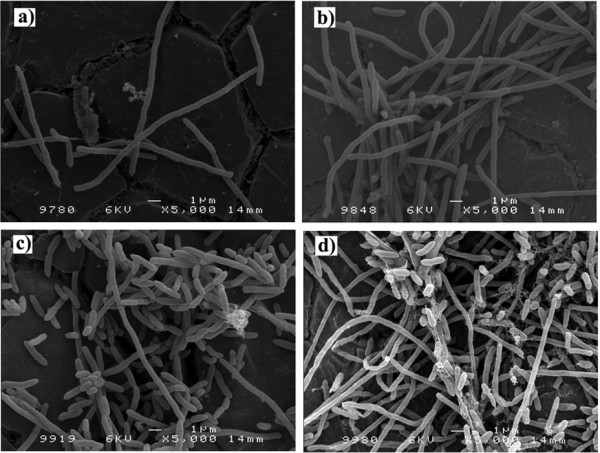
**Dynamic *P. fluorescens *biofilms formed on stainless steel slides with media renewal every 12 h.** FESEM micrographs before phage application: a) 24 h; b) 72 h; c) 120 and d) 168 h old biofilm.

In order to better characterize the effect of dynamic conditions on the biofilm, samples after incubation for 24 to 168 hours were analyzed by field emission scanning microscopy (FESEM) (Fig. 2). The FESEM micrographs show a predominance of filamentous cells in early stage biofilms (24 h and 72 h) and a shift to rod-shape forms in older biofilms (120 h and 168 h).

For the dynamic conditions without renewal of medium biofilm formation was studied only during 24 h and 72 h, as there was clearly decreased biofilm formation ability, most likely due to the lack of nutrients.

Overall, this study shows that the biofilm formation conditions influence the number of cells and biomass present on the coupons as well as the *P. fluorescens *cell morphology. This study is an important basis for the valuation of the effects of bacteriophages.

### Efficacy of phage ϕIBB-PF7A to control biofilms

Single species biofilms do not mimic real conditions, but these biofilms determinedly present advances in the biofilm-phage interaction research field. Here we wanted to investigate how effective the recently isolated T7-like bacteriophage ϕIBB-PF7A [40] is in controlling biofilms under static and dynamic conditions. In these experiments the number of phages applied was 1 × 10^7 ^PFU ml^-1 ^as this concentration is in the range of normally used phage solutions (1 × 10^6 ^PFU ml^-1 ^to × 10^10 ^PFU ml^-1^) [34,37-39].

Initial tests performed with biofilms and phage ϕIBB-PF7A showed that maximum cell reduction was achieved within 4 hours. Therefore, the effect of the phages was followed over a time interval of two and four hours respectively, which is clearly lower than the 24 hours infection period used in other biofilm-phage studies [39,41-43].

As a negative control we also investigated biofilms immersed for four hours in a solution of SM buffer and TSB without addition of phages in order to verify that the observed effects are really related to the phages and not to a detachment of the biofilms as a response to the addition of new SM medium.

**Figure 3:**
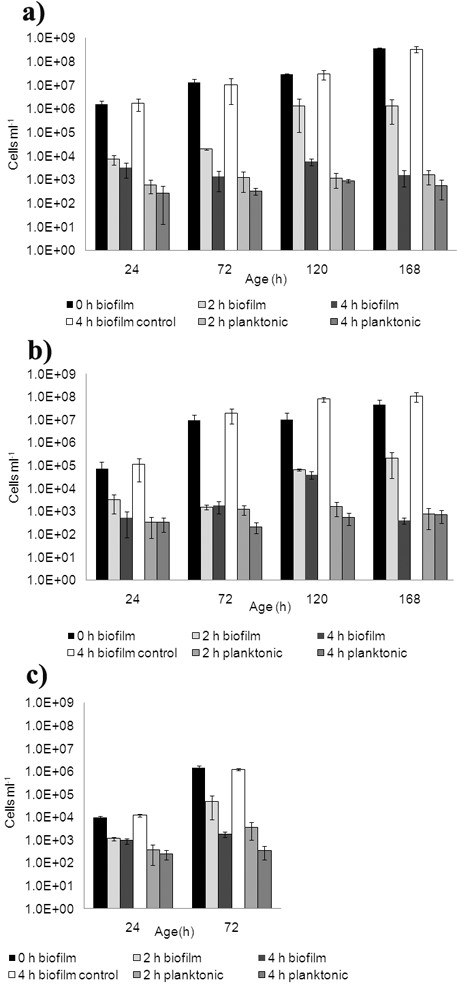
**Number of *P. fluorescens *biofilm and planktonic cells before and after exposure to phage ϕIBB-PF7A.** Cells before phage (time 0 h), control experiments performed for 4 h in SM buffer-TSB, phage exposure for 2 h and 4 h are presented in biofilms were formed under: a) static with media renewal (SR), b) dynamic with media renewal (DR), and c) dynamic with non-renewal of media (DNR). For all experiments n = 6. Error bars indicate standard deviations.

In order to evaluate the phage ability to infect the different *P. fluorescens *biofilms described above, samples were taken after two and four hours for cell and phage enumeration. Already two hours after phage addition, a very strong reduction of the number of cells on the SS slides was observed for all the different biofilms (Fig. 3). In older biofilms (120 h and 168 h) the phage acted somewhat slower, obviously older, stationary phase biofilms delay the cell lysis process. However, under all tested conditions, a 3 to 5 log order reduction of the cell count was detected during only four hours of phage exposure.

Biofilm cell reduction was about one order of magnitude higher in static conditions compared to dynamic conditions, despite of the clearly different cell morphology (compare Figs. 3a and 3b). Furthermore, we also wanted to investigate whether the application of the phages leads to the release of cells. Measurements of cell numbers in the planktonic phase showed that in phage infection experiments there was a detachment of cells or clusters of cells into the liquid medium, although these numbers were quite low, generally below 10^3 ^cells per ml. The measurements of the control coupons show that the biofilms are not detached due to the half-strength TSB and the cell numbers are similar to those at the start of the experiments (Fig. 3) and therefore we conclude that the detachment in phage infection experiments is only due to the presence of phages.

**Figure 4:**
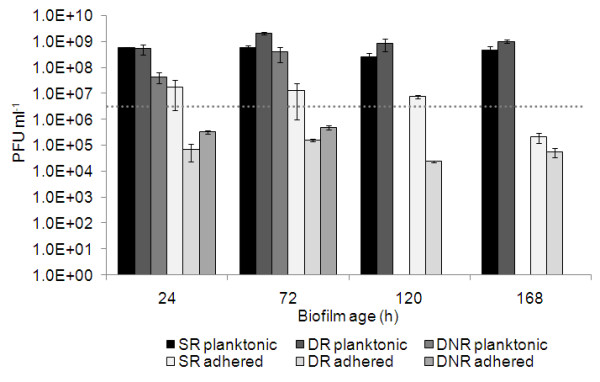
**Number of phages used to infect the different biofilms and progeny phages adsorbed and released after 4 h of infection of biofilms.** Horizontal line: initial number of phages used, static biofilms with media renewal (SR), dynamic biofilms with media renewal (DR); dynamic biofilms without media renewal (DNR). For all experiments n = 6. Error bars indicate standard deviations.

The number of phages in the planktonic phase was approximately the same after infection of the different biofilms (Fig. 4). The main difference in phage counts was related to phages adsorbed to the biofilms and SS slides. A higher number of phages adsorbed to the biofilms and SS slides under static conditions. However, infection under dynamic conditions decreased the ability of phages to adsorb to the substratum and remaining biofilms. Despite this, it was surprising that the number of phages found to be absorbed is approximately in the order of magnitude of the phages applied for the treatment, which indicates a very efficient reproduction of the phages.

**Figure 5:**
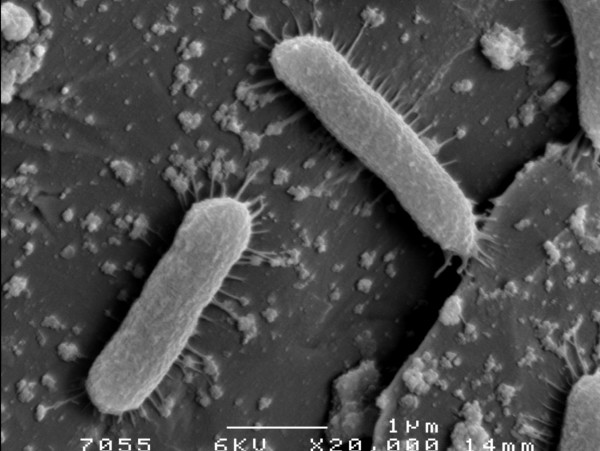
***P. fluorescens*****cells and ϕIBB-PF7A phages on stainless steel slides.** FESEM micrograph taken after infection 30 min of infection of a 24 h static *P. fluorescens *biofilm formed with media renewal every 12 h.

FESEM micrograph taken after 30 min of exposure of a 24 h static biofilm to phage ϕIBB-PF7A shows some individual rod-shaped cells as well as a vast number of phages adsorbed to the SS slides and to the bacteria. The phages appear either individually or as groups of phages (Fig. 5).

**Figure 6:**
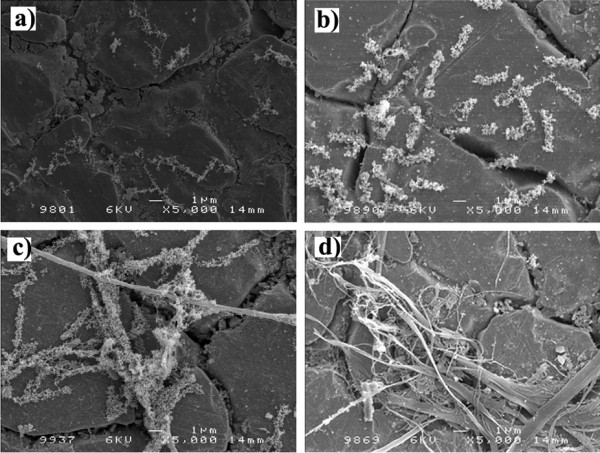
**Cellular debris and biofilm remains on stainless steel slides after 4 hours of phage ϕIBB-PF7A infection of dynamic *P. fluorescens *biofilms formed with renewal of media every 12 h.** FESEM micrographs: a) 24 h; b) 72 h; c) 120 and d) 168 h old biofilm.

Table 1 shows the dry weight on the slides before and after four hours of phage application and the relative biomass decrease after infection of different biofilms. Four hours after infection a considerable biomass decrease was observed for all biofilms. The relative biomass removal accounted for 63 to 91%. In general, older biofilms (120 h and 168 h) showed a lower biomass decrease, most probably due to a higher amount of cellular debris that stayed attached to the SS surfaces (Fig. 6). This cellular debris and dead cells still constitute important sites for phage attachment.

Four hours after phage application, in all experiments, cells from the remaining biofilms were isolated and tested for resistance to the stock phage solution. In none of these tests we found phage-resistant bacteria, indicating that the time of the experiment was not long enough for the bacteria to acquire any form of resistance (data not shown) as described previously by other authors [39,44].

Phage ϕIBB-PF7A is a potential efficient biological agent to control biofilms as the phage's action on biofilms resulted in good biofilm cell log decreases factors and high percentual biomass removal.

## Discussion

*P. fluorescens *is a known milk product contaminant being able to produce extracellular enzymes that spoil milk products. In this work *P. fluorescens *biofilms were challenged with a newly isolated lytic phage. The data presented here, shows the potential of the novel phage ϕIBB-PF7A [40] for controlling and reducing the *P. fluorescens *biofilms formed under three different conditions. Phage ϕIBB-PF7A was able to reduce greatly the number of biofilm cells present on stainless steel slides already after 2 h of infection experiment and this reduction was even more noticeable after 4 h of phage application. The biofilm cell log reduction factors, after 4 h of phage exposure varied between 3 and 5. Complete eradication was not achieved in any of the studied cases, however it is evident that phage ϕIBB-PF7A can greatly decrease the number of biofilm cells present on the surfaces. Similar cell log reductions have been reported in other biofilm-phage experiments, however in all these other studies the phage infection period used was 24 h [39,41-43]. This shows that phage ϕIBB-PF7A has very good potential as a biofilm control agent as it can achieve the same result in only 4 hours.

In this study, the formation of biofilms on substrata immersed on microplates does not imitate true conditions observed in a variety of environments; nevertheless it is a simple, rapid and reproducible method with which it is easy to assess the influence of different parameters. Biofilms formed show that *P. fluorescens *are able to grow either as rod-shaped or as filamentous like form. This change in morphology is due to the rotation applied (100 rpm) to form dynamic biofilms. However, there is a clear switch on the cell morphology: young biofilms (24 h and 72 h) are predominantly filamentous like, while older biofilms (120 h and 168 h) consist of more rod-shaped cells (Fig. 2). Unlike in *P. aeruginosa *and *P. putida *where this filamentous cell morphology has already been described [45-47], a filamentous morphology has not been reported for *P. fluorescens*.

Biofilm cell lysis starts faster under dynamic than under static conditions, but interestingly, the total relative biofilm reduction after four hours was not as effective in dynamic biofilms compared to static biofilms. Under dynamic conditions, phages probably meet their hosts faster than on static conditions, indicating the important role of the convection mechanism. Conversely, under static conditions, the lack of agitation keeps the progeny phages in the proximity to other neighboring biofilm cells. Under static conditions, the new phages, which are released due to phage infection and lysis of the host cells, are not transferred to the bulk and this enhances the biofilm cell lysis. This hypothesis is confirmed by the values of adsorbed phage counts which were more than two orders of magnitude higher under static conditions compared to dynamic conditions (~1 × 10^7 ^and <1 × 10^5 ^PFU ml^-1 ^respectively). In both situations the phage numbers in the planktonic phase were approximately the same (1 × 10^9 ^PFU ml^-1^). Nevertheless, it is important to state that the same efficiency of cell lysis was reached within four hours after phage infection under dynamic and static conditions.

Biofilm formation is directly related to the supply of nutrients [48-52]. In this work, *P. fluorescens *biofilm formation under non-renewed media conditions was the worst and least effective method for producing biofilms (Fig. 1). This lower ability of forming biofilms might be due to depletion in nutrients available for the sessile bacterial cells, which were consumed by the planktonic cells rendering difficult the multiplication of the biofilm bacteria. Cerca et al. [53] observed also this enhanced biofilm formation under fed-batch (nutrient renewal) conditions in comparison to batch (without nutrient renewal) formation of *S. epidermidis *biofilms. The efficiency to remove biofilms by phages is decreased in biofilms where a change of the medium was not performed. There may be various reasons, including a decreased number of daughter phages and longer phage development cycles in starving bacteria [32,54], but also less efficient phage adsorption.

Biofilms are composed of pores and channels [55] through which nutrients reach cells present on different layers. It may be also assumed that phages are able to circulate through these channels and pores and by this way reach and adsorb to cells on different biofilm layers, including the basal layer of the biofilm. This study shows that phage application to biofilms resulted in a release of cells to the planktonic phase. This is most likely due to the infection of cells at different layers which causes the sloughing off of parts of biofilms to the liquid medium. After the release of these biofilm clusters to the planktonic phase, the detached biofilm cells are most likely attacked by phages present on the liquid medium as in all situations the number of cells detectable on the planktonic phase was always bellow 10^3 ^cells ml^-1^.

It has been reported that phage aggregation and fixation on surfaces is a mechanism of protection that phages adopt as a strategy of survival from inactivating environmental stresses [55-58]. In this work it is shown that phage ϕIBB-PF7A in fact adsorbs to stainless steel and phages are found individually or in aggregates (Fig. 5). Phage adsorption was clearly dependent on the infection conditions applied. Static conditions allow the deposition of phages while dynamic conditions suppress phage fixation.

Coexistence of phages and bacteria has frequently been reported and discussed in relation to bacterial resistance of the bacteria to the phages as well as a consequent mutation of phages [59]. In this work, phage action on the biofilms formed was studied merely for 4 h which was not a period long enough for the emergence of resistance. Possibly longer periods of phage action on the biofilms would result in the appearance of phage resistant bacteria.

Tail et al. [39] has obtained complete eradication of 24 h biofilms of *E. cloacae *when exposing the biofilms during 24 h to a solution of 3 different phages. Curtin and Donlan [31] have adopted a different approach of phage application in order to combat *S. epidermidis *biofilm formation onto catheters. Instead of treating biofilms once they were already present, these authors have adopted pre-treatment of catheter surfaces with phages to decrease bacteria adhesion to the surface. In food industry, pre-treatment of all surfaces is more complicated, so the combination of phages in a cocktail to achieve biofilm eradication is more realistic. Furthermore, there might be attractive scenarios where phage sanitation is applied before or with chemical biocides, which may make sanitation more efficient and therefore decreasing the necessary amount of chemicals.

Phages have been suggested as sanitation agents for different bacteria present in food products and industrial facilities [27-30,55]. For instance, LISTEX™P100 from EBI Food Safety and LMP-102 from Intralytix were both approved in 2006 by the FDA to combat *Listeria monocytogenes *present in foods and to eliminate *L. monocytogenes *from industrial surfaces. The phage used in this study, ϕIBB-PF7A, is an interesting biological agent as it has a great ability of lysing biofilm cells in an exceptionally rapid time.

## Conclusion

Phage ϕIBB-PF7A may prove to be useful for the biocontrol of *P. fluorescens *strains in dairy and other food related industries. In laboratory studies it was highly efficient in reducing the number of cells present even in mature *P. fluorescens *biofilms, formed under different conditions, which persuades us to continue further biofilm and phage studies including studies with mixed biofilm communities.

## Methods

### Bacteria and growth conditions

*P. fluorescens *was isolated from a Portuguese dairy plant located in Paços de Ferreira and grown at 30°C in Tryptic Soy Broth (TSB, Fluka). Solid TSA medium contained 1.2% w/v of Bacto™ agar (Difco) and the soft agar top-layer contained 0.6% of Bacto™ agar. All bacteria were subcultured once and glycerol stocks were done and stored frozen at -80°C until further use.

### Bacteriophage isolation

Bacteriophage ϕIBB-PF7A was isolated from a sewage treatment plant in Esposende (ETAR de Esposende, Portugal). This phage was chosen amongst different *P. fluorescens *phages isolated as it was able to infect a higher number of *P. fluorescens *isolated from a dairy industry [40].

### Bacteriophage propagation and concentration

Concentrated phage solutions were produced using the plate lysis and elution method as described by Sambrook & Russel [60] with some modifications. Briefly, a top agar was prepared containing 1 ml of phage solution and 1 ml of a bacterial overnight culture in 30 ml of soft-agar. This agar was added to 250 mL T-flasks with a thin bottom layer of TSA. After solidification of the top agar layer the T-flasks were incubated at 30°C overnight. Afterwards, the flasks were eluted with SM buffer and the phage lysate was first concentrated with PEG 8000 and then purified with chloroform. Samples in SM buffer were stored at 4°C until further use.

### Phage titration of stock solution

Bacteriophage titer was analysed as described by Adams [61]. Briefly, 100 μl of diluted phage solution, 100 μl of a bacterial overnight culture, and 3 ml of molten agar were mixed in a glass tube and poured into a TSA containing Petri dish. Plates were incubated for 18 h after which plaque forming units (PFU) were counted.

### Biofilm formation

Biofilms were formed on stainless steel (SS) 1 × 1 cm slides for different time periods (24 h up to 168 h) according to the method described by Cerca *et al*. [53] with some alterations. Briefly, SS slides were placed on the wells of a 6 well microplate containing each well 6 ml of TSB medium. Bacterial culture (50 μl) with an OD_600 _of 1.0, which corresponds to approximately 1.79 × 10^9 ^cells ml^-1^, was added to each well and the microplate was incubated at 30°C under different conditions. Under static conditions, the 6-well microtiter plates were put in an incubator and the biofilms were formed without agitation and with a change of medium every 12 h during the whole duration of the experiment. Under dynamic conditions, the 6-well microtiter plates were put on an orbital shaker at a constant speed of 100 rpm and two different biofilm formation strategies were studied – with the change of medium every 12 h and without the change of medium. Abbreviation will be used for the different biofilm formation conditions: static conditions with media renewal every 12 h (SR), dynamic conditions with media renewal every 12 h (DR) and dynamic conditions without renewal of media (DNR).

### Biofilm infection

Biofilms were allowed to form on stainless steel (SS) slides for different times and conditions in 6-well microplates. Afterwards, the SS slides with biofilm were immersed twice in fresh PBS and placed in new microplates with 3 ml of TSB and 3 ml of phage solution with a concentration of 10^7 ^PFU ml^-1^. The 6-well microplates were incubated at 30°C the same conditions at which the biofilms were formed. Control experiments were performed at the same conditions with the SS slides put, after immersion in PBS, in new microplate wells with 3 ml of TSB and 3 ml of SM buffer. Biofilms before and after phage infection were analyzed for number of cells (CFU counts) and phages (PFU counts) attached to the surfaces and on the planktonic phase as well through dry weight determinations (see below).

### CFU and PFU counts of biofilm samples

The number of bacteria and phage present on the SS slides before and after infection of biofilms formed under different conditions was enumerated in order to estimate the efficiency and adsorption characteristics of the phage. Therefore SS slides with biofilms were washed twice by immersion in PBS and afterwards put in 50 ml tubes containing 6 ml of 0.9% saline solution. The tubes were thoroughly mixed (vortexed 4 × 30 sec) and serial dilutions were immediately performed in 0.9% saline solution for CFU counts and in SM buffer for PFU counts. For CFU counts the samples were immediately plated on TSA plates and for PFU counts samples were immediately plated using the method described above for phage titration. Six independent parallels were performed for the different countings.

### CFU and PFU counts of planktonic samples

Samples for planktonic CFU and PFU counts were removed from the 6-well microtiter plates and serial dilutions were performed. All samples were immediately processed. Six independent parallels were performed for the different countings.

### Biofilm dry weight determination

Biofilm dry weight determinations were performed as described by An & Friedman [62]. Briefly, stainless steel (SS) slides with biofilms formed under different conditions were removed from the microplates and rinsed by immersion in fresh PBS. Afterwards, the slides were dried at 100°C for 24 h and weighed. The SS slides were then carefully washed, dried again for 24 h at 100°C and weighed (empty control). Biofilm dry weight was calculated from the difference between these measurements. Biofilm dry weight determinations represent six independent parallels for controls (time 0 of infection) and for phage treated slides (4 h after infection).

### Resistance assays

Biofilm cells that remained on the stainless steel surfaces were analysed for resistance as described by Sillankorva et al. [33] with some alterations. Briefly, swabs were used to collect the bacteria and put on tubes containing 1 ml of saline 0.9%. After, dilutions were made and plated on TSA dishes. After overnight incubation, 20 colonies from different dishes were picked and grown on flasks with 50 ml TSB medium for 10 h. Bacterial lawns of these cultures were done and tested for resistance using the spot test with the phage stock solution. The dishes were incubated for 18 h at 30°C and checked for presence of phage plaque.

### Field Emission Scanning Electron Microscopy (FESEM)

Samples were taken before phage infection and after 4 h of phage infection. The SS slides were rinsed by immersion in fresh TSB media before adding 2.5% glutaraldehyde and incubation at 4°C for 1 h. Dehydration was carried out with an ethanol series from 30% to 50% to 70% to 80% to 90% and absolute, followed by critical drying (Critical Point Dryer CPD 030). Biofilms were coated with platinum coating and analyzed with FESEM in a JEOL JSM-6300F (Tokyo, Japan) instrument.

## Authors' contributions

SS performed all practical experimental work and wrote the manuscript. PN contributed with his experiences in phage cultivation and JA with her competence in biofilm control. JA and PN supervised the work. The final manuscript was read and accepted by all co-authors.
